# Prevalence and risk factors associated with female anal sex in the context of HIV/AIDS in the selected districts of Tanzania

**DOI:** 10.1186/s13104-017-2452-9

**Published:** 2017-03-27

**Authors:** Elizabeth H. Shayo, Akili A. Kalinga, Kesheni P. Senkoro, Judith Msovela, Erick J. Mgina, Angela E. Shija, Godlisten Materu, Stella P. Kilima, Leonard E. G. Mboera, Julius J. Massaga

**Affiliations:** 1 National Institute for Medical Research Headquarters, P.O. Box 9653, Dar es Salaam, Tanzania; 2 Tukuyu Medical Research Centre, P.O. Box 538, Tukuyu, Tanzania

**Keywords:** Risk factors, Practices, Female anal sex, HIV/AIDS, Tanzania

## Abstract

**Background:**

Female anal sex is a receptive type of sexual practice among heterosexual couples where the penis is inserted into the anus of a female partner. In the Western world, a number of studies and interventions have been carried out on anal sex among men due to its potential risks to HIV transmission. In African countries, including Tanzania, there is dearth of information on the risks inherent in practices associated with female anal sex in the general population. The objective of this study was to determine the prevalence and risk factors associated with female anal sex in fuelling HIV transmission in selected districts of Tanzania.

**Methods:**

This study was conducted in four districts of Tanzania of Kinondoni, Tanga Urban, Makete and Siha. Both quantitative and qualitative methods i.e. household interviews and focus group discussions were employed in data collection. Study participants included community members of aged 15 and above such as heads of the household, adolescents, bar workers and commercial sex workers.

**Findings:**

A total of 903 individuals were interviewed, 60.6% of whom were females. When respondents were asked to indicate whether they had ever been tempted to practise FAS, 167 (18.5%) reported to have been tempted in the past 12 months. Of these, 44 (26.3%) respondents had at least practised FAS. Risky practices associated with FAS were forced sex, multiple partners, frequency of engaging in FAS, low use of condoms during FAS, low rates of HIV testing among partakers, poor perception of the risks to acquire HIV through FAS and use of lubricants.

**Conclusions:**

In this population, the frequency of FAS practice was rather low. And yet, FAS practice attendant risk factors are likely to exacerbate HIV transmission. As such, there is a need for further exploratory studies to determine and document drivers of FAS. In addition, public health education should be provided with regard to the risks of contracting HIV associated with FAS practices.

**Electronic supplementary material:**

The online version of this article (doi:10.1186/s13104-017-2452-9) contains supplementary material, which is available to authorized users.

## Background

Anal sex has been identified as a predictor of HIV infections among women [1] as it was also revealed among the men who had sex with men (MSM) [2, 3]. Indeed, anal sexual intercourse is more vulnerable to tears and the larger surface of rectum provides more opportunity for viral penetration [4, 5]. A study conducted in the United States revealed that per contact probability of HIV transmission has been estimated to be 10 times higher for penile anal than penile vaginal sex [6] as it was also reported by the European Study Group on Heterosexual Transmission of HIV [7] Anal sex practice among sexually active women is growing and globally is estimated to be about 5–10% [8]. There is a need to investigate the prevalence of female anal sex practices and the associated risk factors to HIV infection in order to inform policy makers to take appropriate action.

Although anal sex practices have been researched in the US for more than two decades [9, 10], it was only recently done in some parts of Africa [11]. A study in South Africa revealed that among female commercial sex workers, 42.8% had practiced anal sex and the prevalence of HIV infection was 61.3% compared to 42.7% for those who engaged in other sexual practices [12].

In Tanzania available reports show that apart from penile vaginal intercourse, female anal sex is also practised in more than 40% of female sex workers [13] and 8% of adolescents [11]. The HIV infection risks of anal sex in a study in Dar es Salaam have also been documented [14].

Despite anal sex generally being acknowledged as the riskiest factor for HIV infection, it is its associated practices that heighten the inherent risk of HIV infection even further. These practices include unprotected receptive anal intercourse, multiple sex partners, inconsistent condom use, lack of knowledge about HIV risk, and negative or complacent attitudes toward safer sex [15]. As it has been reported in science daily, those using lubricants were three times more likely to have rectal sexually transmitted infections (STIs) as many of the products were toxic to cells and rectal tissues [16]. Socio-cultural factors, social stigma and discrimination, may also lead to increased risk of HIV infection in females practising anal sex as it was also found among MSM [17]. In Tanzania, there is dearth of information on the prevalence and risk factors to FAS that are associated with HIV transmission. This study was, therefore, carried out to determine the prevalence and risk factors associated with female anal sex in the context of HIV transmission in selected districts of Tanzania.

## Methods

### Study area

The study was undertaken in four districts of Kinondoni, Tanga Urban, Makete and Siha. Kinondoni district is located in the northern part of Dar es Salaam City with a population of 2,497,940. The Kinondoni Municipal Council medical care coverage plan comprises 33 public and 168 private health facilities. Tanga district is the headquarters for Tanga region (province) and one of the major ports in Tanzania. The town has a great potential of attracting immigrants from rural areas as it has employment. It has the estimated population of 261,613. Tanga has 52 health facilities both public and private. Kinondoni and Tanga are located in the coastal area where anal sex among men is believed to be practised to a larger extent with the highest HIV prevalence than that of the general population [18]. The two coastal districts were selected due to the proximity with regions previously documented to have high rate of MSM [19, 20]. Therefore, there was a possibility to observe similar trend of FAS in the selected sites. Makete district, on the other hand, is one of the five districts of Njombe region of Tanzania. It is bordered to the North and West by the Mbeya region to the East by the Njombe district and to the South by the Ludewa district. The district has an estimated population of 97,266. It has 30 health facilities both private and public. Siha district is one of the seven districts of Kilimanjaro region. The district shares borders with Hai district to the South, Longido and Rombo districts to the North West, Meru and Hai districts to the South East. The district covers about 1168 sq. The district has a population of 116, 313 (Tanzania Census report, 2012).

### Study design and population

This was a descriptive cross-sectional study that employed both qualitative and quantitative methods. The study targeted individuals aged 15 and above who had lived in the study areas for more than 3 consecutive years. They included general community members (adults and youths), high risk groups such as bar workers and commercial/street sex workers.

### Sampling and recruitment of informants

A total of four districts were sampled purposively using the criteria of HIV-prevalence and existence of anal sex practices. Makete and Siha were selected from inland regions with high and low HIV-prevalence, respectively. Kinondoni and Tanga were selected from regions along the coastal belt and known to have anal sex practices among men. Two wards were randomly selected from each district. Finally, two villages were selected from each ward. Households were randomly selected from the respective villages and in each selected households one individual aged ≥15 years old was chosen for an interview. To maintain gender balance, researchers conducted interviews by alternating between females and males, and youths and adults, throughout the survey. Individuals who had a short stay and those who were found to be sick on the day of the interview were not interviewed. Commercial sex workers (CSWs) and bar-maids, and some adults (males, females, pregnant mothers) and youths were conveniently recruited for the qualitative component of the study.

### Data collection

#### Household surveys

Face-to-face interviews were held with the selected members of the households using a structured questionnaire. The interviews were conducted in the household premises of the respondents. Quiet places were chosen by the respondent within the household premises to ensure enough privacy and freedom of expression because of the sensitive nature of the topic. A single interview lasted about 30–45 min. The study elicited information on the magnitude of heterosexual anal sex practices among females of different population groups, community awareness about female anal sex practices, common practices involved in female anal sex, perceived risk factors to FAS associated with HIV infection, and drivers for female anal sex practices in the community. To ask such sensitive questions, the interview started by asking the respondents general questions about their knowledge on HIV/AIDS and their awareness of different sexual practices.

#### Focus group discussions

A total of 20 focus group discussions (FGDs) for different age groups and sex—adult females, adult males, female youths, male youths, bar-maids and pregnant mothers from each district—were held. Discussions were held separately for each group without mixing the sexes or age groups as categorised above to enhance freedom of expression. Participants in each group ranged from 8 to 12 and each discussion was facilitated by an experienced social scientist. The venues for the discussions were in school premises or village offices as there were enough spaces to enhance privacy, confidentiality and freedom of expression. The discussions were tape-recorded after obtaining consent from the participants. The FGDs facilitated the generation of Additional file 1 to complement household survey data and gain a detailed understanding of issues that were difficult to capture in the interviews. Each FGD lasted for about 1.5–2 h. Since both the household survey and FGDs were conducted concurrently, there was little chance for participants to participate in both activities.

### Data analysis

Quantitative data were double entered using EPIDATA (version 3.1) software. Data from the structured interviews were analysed using SPSS (version 16) and STATA. Variables were tested at 5% level of significance; *t* test and Chi square were used to establish association among the variables. Qualitative data transcriptions was done verbatim and thereafter analysed manually to detect relevant themes and develop sub-themes. Consequently, texts with similar meanings were placed in the respective sub-themes and quotation that reflected informants own words were taken, some of which were presented verbatim to strengthen the qualitative narrative presentation. Eventually, the quantitative and qualitative data were triangulated to boost the trustworthiness and validity of the data generated and analysed.

## Results

A total of 903 respondents were interviewed from four study districts, from both rural and urban settings. Males accounted for 39.4% and females for 60.6%, with little variation across districts. The majority of the respondents (80%) were aged below 45. The mean age of the respondents was 33 ± 12.5 (range = 15–84). Slightly more than half (52.9%) of the respondents were married, and most of them were from Makete. About 59.9% had primary school education; district wise, Makete had few individuals with secondary and college education and many with no formal education compared to other districts (Table 1).

**Table 1:** Socio-demographic characteristics of the respondents (N = 903)

	Siha (%)	Tanga (%)	Kinondoni (%)	Makete (%)	Total (%)
Sex					
Male	73 (20.5)	94 (26.4)	97 (27.3)	92 (25.8)	356 (100)
Female	153 (28.0)	133 (24.3)	128 (23.4)	133 (24.3)	547 (100)
Age					
<24 years	57 (23.5)	91 (37.6)	49 (20.3)	45 (18.6)	242 (100)
25–34	72 (23.7)	76 (25.0)	83 (27.3)	73 (24.0)	304 (100)
35–44	49 (27.7)	31 (17.5)	38 (21.5)	59 (33.3)	177 (100)
>45	42 (27.1)	23 (14.8)	42 (27.1)	48 (31.0)	155 (100)
Mean age (SD)	33.8 (13.1)	29.3 (11.1)	34.1 (12.4)	35.4 (12.4)	
Marital status					
Single	81 (27.6)	98 (33.3)	89 (30.3)	26 (8.8)	294 (100)
Married	110 (23.0)	101 (21.1)	102 (21.4)	165 (34.5)	478 (100)
Divorce/separated	13 (14.7)	14 (21.2)	16 (24.2)	23 (34.9)	66 (100)
Widowed	22 (33.9)	14 (21.5)	18 (27.7)	11 (10.9)	65 (100)
Education					
No formal education	37 (34.9)	13 (12.3)	10 (9.4)	46 (43.4)	106 (100)
Primary school	136 (24.9)	133 (24.4)	120 (22.0)	157 (28.7)	546 (100)
Secondary school	47 (22.0)	76 (35.5)	75 (35.0)	16 (7.5)	214 (100)
College/University	6 (16.2)	5 (13.5)	20 (54.1)	6 (16.2)	37 (100)

### Heterosexual female anal sex practices

Out of the 903 respondents, 167 (18.49%) reported to have been tempted/coerced into engaging in female anal sex in the past 12 months. Some 52% of these respondents happened to be male. Both in quantitative and qualitative data, the findings show that male participants were more tempted to engage in anal sex with females than vice versa. Also, such a tendency was found to be more pronounced in urban than in rural areas. Out of these, 44 (26.35%) accepted to do so (Table 2). Binomial probability test was used to compare the proportion of respondents who were tempted and actually practised anal sex between coastal and inland districts. Significantly more respondents from Tanga and Kinondoni (Coastal districts) were tempted and practised anal sex than the inland districts of Siha and Makete (p < 0.001). Out of the 44 respondents who reported to practise FAS 41% were from Kinondoni, 30% from Tanga, 27% from Makete and a mere 2% from Siha district. Using Makete district as a reference point, comparison on the practice of FAS was carried out between districts using one sample proportion test (Table 2). There was a significantly high difference between Siha and Makete and less significance difference between Kinondoni and Makete and no difference between Tanga and Makete. However, when the analysis was done within districts by comparing those who were tempted and those agreed to go ahead and engage in female anal sex, Makete district had the largest proportion of individuals who accepted to practise anal sex. According to the focus group discussion, female participants in the coastal districts would approximate a 50% prevalence of anal sex whereas males-adults and boys would approximate the prevalence to be as high as 80%.

**Table 2:** Proportions of respondents who were tempted and practiced female anal sex by district

District	Total participated (N)	% convinced	Total convinced (n)	% practiced
Siha	226	4.9	11	9.1
Tanga	227	31.7	72	18.1
Kinondoni	225	27.1	18	29.5
Makete	225	10.2	12	52.1
Overall	903	18.5	44	26.4

### Risk factors towards FAS practices are presented below

Cultural beliefs were said to be the main reason behind more females demanding anal sex than males as the following verbatim statements illustrate: “*You know if the penis enters the anus and get in touch with faeces it is just like going to traditional healers. The male partner will never leave you. It is like you have already bewitched him”* (Pregnant woman, Kinondoni); “*For those in marriage, FAS is one of the things to satisfy their husbands. Married women do speak for themselves that once you give your husband anal sex he will never perform extra marital sex*” (men FGD Tanga).

### Forced FAS

Among those who practised FAS, four females reported to have been forced by their regular partners and five by their casual partners (with no prior relationship), and this was supported by the qualitative findings; “*Women are forced to engage in anal sex by their husbands, especially when they are drunk,*” FGD men, Kinondoni. Conversely, two males reported to have been forced by their casual female partners and guardians to engage in anal sex with them. In other words, the source of inducement could come from either gender. Respondents also revealed that force (coercion) was applied to most of these individuals after the initial seduction attempts had failed. In the qualitative study, men were forced by circumstances to practice anal sex with women to satisfy their sexual arousal and maintain their manhood as the following statement reveals: “*In this area if you won’t perform anal sex you are perceived as if you are not from Tanga. They regard you as an intruder… Our women in this area if you sex with them without going anal they don’t get satisfaction. They can even declare before their friends that you know nothing about how to make love*” (FGD men, Tanga). More qualitative evidence emerged from Tanga to affirm the widespread nature of the practice in some quarters of the town: “*…we*—*youths*—*are so affected by the anal sex practice that nowadays even girls treat anal sex as a normal practice*” (FGD boys, Tanga).

### Number of partners

**Fig. 1:**
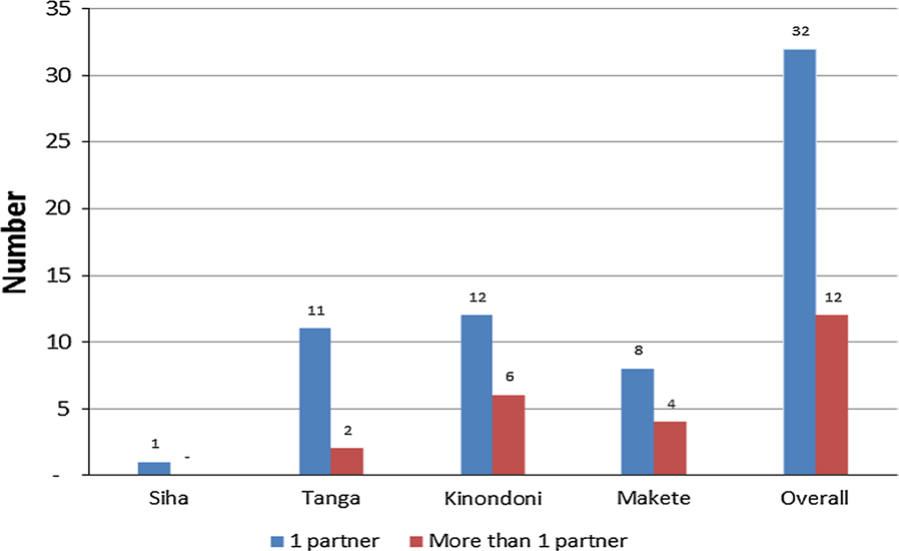
Number of partners among FAS group (Upcountry = inland)

Out of 44 respondents who practised FAS, 72.7% and 27.3% reported to have regular and casual partners, respectively, for the past 1 year (Fig. 1). However, 50% of those who practised FAS in Kinondoni and Makete had more causal partners. Out of those who had ever been persuaded to practise anal sex, 59.8% reported that casual partners were behind the temptation. Regular partners were also reported by about one-third (30%) of the participants as ever having tempted their spouses to engage in anal sex. Out of these, 18% were in marital relationship.

### Frequency of female anal sex

**Fig. 2:**
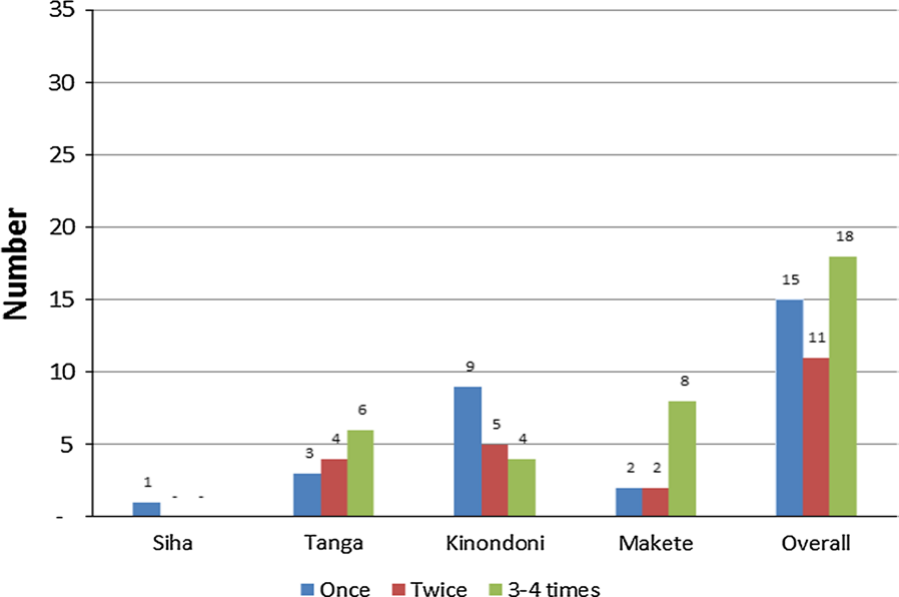
Reported frequency of female anal sex per year

A good number of individuals engaged in FAS (18/44) had performed anal sex in heterosexual relationships for more than three times in a year. Although the variation existed between districts regarding the frequency of performing FAS, Makete seem to have a large proportion of individuals who had practised anal sex for more than four times in a year (Fig. 2).

### Use of condom

**Fig. 3:**
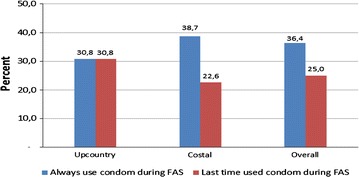
Proportion of condom use by respondents during performing FAS (Upcountry = inland)

A notable proportion of the respondents (36.4%) who reported to have ever practised FAS revealed to have always used condoms; whereas 25.0% used them during the last episode (Fig. 3). However, there was no significant difference in condom use among respondents practising FAS between the districts (p value 0.75). Several factors were mentioned to contribute to non-use of condoms. Reduction of sexual pleasure was mentioned by the majority in the qualitative study. Other reasons included unavailability of condoms nearby, inconveniences when inserting the penis into the anus, and some did not use a condom to as a gesture of loyalty to the partner or as a sign of fidelity. In fact, many of the FGD discussants said that many of the people who practised anal sex did not use any form protective measures against HIV. This non-condom use is further illustrated by the following statements: “*For anal sex it is difficult to use any preventive measure. This is because the anus is so dry and has more heat that causes the condom to tear. So the safety of using condoms is so minimal”* (FGD youths, Kinondoni); “*Lubricants are always used in anal sex; therefore, it is also difficult to use condoms in the anus because of its nature. Otherwise a person should be well experienced in doing FAS. Special types of jelly are used by commercial sex workers and these can’t be used concurrently with condoms*” (FGD Girls, Tanga).

### Perceived level of risks to acquire HIV infection among those practicing female anal sex

Those who practised anal sex were asked about the perceived risk of acquiring HIV infection during anal sex in heterosexual intercourse. Over half (56.8%; 25/44) of the respondents who engaged in FAS reported to have no risk at all of acquiring HIV/AIDS. Of those who reported to be at risk, 10 (38.5%) perceived themselves to be at moderate risk, 5 (19.2%) at high risk and 4 (15.4%) at low risk. Although more than 50% of the respondents in Makete who were tempted to perform anal sex agreed, only a small proportion perceived doing so subjected them to the risk of acquiring HIV infection compared to other districts. A good number rated moderate risks and one of the respondent reported to be already HIV infected.

FGD participants in all the groups said that when individuals engaged in anal sex there is a likelihood of getting physical injuries due to severe friction attributed to the tightness and dryness of the anus. They further explained that this state of affairs contributes greatly to the acquisition of infection including HIV if one of the partners is infected: “*There is a danger of acquiring HIV infection because the place where penis is inserted is much tighter, so during the process the anus is torn or the penis can get bruises. When there is blood contact from both partners, HIV is directly transmitted if one of partner is infected*” (FGD Female participant, Kinondoni).

Although the majority in the FGDs perceived the risk to acquire HIV infection to be high in FAS practice, few participants failed to see such a link. Few participants from Siha district reported there being no direct relationship between anal sex and HIV transmission. The reasons given were: first HIV virus stays in vaginal fluids, hence making it difficult for the virus to stay in dry places like anus; second anal sex is practised only by a small proportion of the population and so there is a lower risk to acquire infection than during vaginal intercourse which is practised by the majority of heterosexual partners.

### HIV testing

Out of the 903 respondents, more than 70% had gone for HIV testing. A larger proportion of individuals tested following the death of the partner (45.5% and 63.2% periphery and coastal districts, respectively), and the differences were significant (p value <0.001). A similar trend was observed among anal sex group whereby 30/44 tested for HIV, of which 56.7% opted to test after the death of their partners (Table 3). Other reasons for testing included its being a requirement for marriage, following advice from care providers and being forced to undertake one due to an elongated period of sickness.

**Table 3:** Reasons mentioned for undergoing HIV testing among FAS group

Districts	During pregnancy no (%)	Campaign no (%)	Death of a partner no (%)	Others no (%)	Total no (%)
Inland	0 (00)	1 (9.09)	5 (45.5)	5 (45.5)	11 (100)
Coastal	3 (15.8)	2 (10.5)	12 (63.2)	2 (10.5)	19 (100)
Total	3 (10.0)	3 (10.0)	17 (56.7)	7 (32.3)	30 (100)

### Lubricants used

Out of 903 respondents, 302 (33.44%) were aware of the lubricants used in FAS, oil being the main one. Similar findings recurred in qualitative findings whereby the majority of FGD participants mentioned petroleum and K-Y jelly as the most commonly applied lubricants during anal sex intercourse to facilitate penile penetration. K-Y jelly was mostly mentioned in Kinondoni and Tanga. K-Y jelly was described as rather too expensive for many of the respondents to afford and hence was mainly accessible to the high income group. Others would use any type of substances such as soap, oil, and saliva, whose quality and safety remain unknown and untested: “*Others tend to use condoms as they are believed to have oil which helps to soften the anal canal. They also fear of getting infections*” (FGD women, Kinondoni).

In the household survey, several ways were used for lubrication of the anus before intercourse. These include the use of skin oil being mentioned by the majority of the respondents (Table 4). The use of lubricants was seen important because the anus does not produce fluids as the vaginal after sexual arousal or stimulation: “*A high proportion of individuals performing FAS usually use lubricants because the romance only stimulates the vagina that at the end produces fluids/mucus but in the anus there is nothing like that. That is how God created*” (FGD, girls Tanga).

**Table 4:** Proportion of respondents who mentioned lubricants that facilitate anal sex (N = 302)

Districts	Condoms no (%)	K-Y jelly no (%)	Other oils no (%)	Saliva no (%)	Total no (%)
Inland	19 (22.62)	0 (0.00)	45 (53.57)	4 (4.76)	84 (100)
Coastal	15 (6.88)	18 (8.26)	155 (71.10)	15 (6.88)	218 (100)
Total	34 (11.26)	18 (5.96)	200 (66.23)	19 (6.29)	302 (100)

## Discussion

The study was aimed at determining the prevalence and risk factors associated with female anal sex in fuelling HIV transmission in heterosexual liaisons. The study findings have revealed that although the reported magnitude of FAS remained low (26.3% of those who were coerced), this practice seems to be under-reported by interviewed respondents. The practice seems to be widely practised especially in coastal belt districts as reflected in qualitative findings. The low prevalence of anal sex was also revealed in a study conducted in Ethiopia where only 4.3% out of 154 respondents had engaged in anal sex [5]. Despite the small magnitude of anal sexual intercourse, it is equally important to make a follow up as this practice is the riskiest sexual practices in HIV transmission. In fact, many individuals in the study reported being convinced to engage in FAS but the receptive rate was higher in Makete than in the three other districts under review. Some interpretation can be drawn from these findings: the lowest acceptance level in Kinondoni implies that the respondents are more aware of the dangers associated with FAS and know how to negotiate their way out of tricky situations when sexual partners demand for FAS. The acceptance rate in Makete can be well correlated with their lower level of education, compared to other districts as the study findings revealed. Moreover, the findings reveal that males were more tempted to engage in FAS than females. This finding attests to the consistency with the data obtained from the qualitative section. Generally, based on prior assumptions, we expected to find more females to be tempted than males as far as African dominantly patriarchal culture is concerned (for example, African women cannot seduce). As the reverse was true, this finding suggests a need for more health education in both districts taking into considerations that Makete district still has and is still reeling from the effects of a high prevalence of HIV infection. A recent study done in Kenya found that, apart from penile-vaginal intercourse, more than 40% of female sexual workers reported ever practising anal sex and 36.1% had experienced dry sex [13]. A study conducted in Tanzania found self-reported anal sex among adolescents and youths to stand at about 8% [11]. To our knowledge this is the first study in Tanzania that has explored specifically female anal sex in heterosexual relationships and its associated practices in the general population.

Several health risks practices that are associated with FAS and the danger of acquiring HIV infection were revealed in this study. Having multiple partners was mentioned as one of the factors, with coastal districts reported to have a large proportion of individuals engaged in FAS, especially casual ones. Although multiple partners were linked to all sexual practices, it is a risk factor to acquire HIV infection especially for those who engage in FAS because the rectal tissues are much more vulnerable to tearing during intercourse and the larger surface area of the rectum/colon provides more opportunity for viral penetration and infection than the virginal intercourse [21]. A previous study has revealed that the majority of students who reported having had anal sex also had multiple sexual partners and most of them had not used condoms during such anal sexual intercourse [11, 22, 23]. Another study conducted in Ethiopia revealed that, although the proportion of oral and anal sex appears to be low, the proportion of youth engaged in multiple sexual partnerships, and the extremely low and inconsistent use of the condom during such sexual encounters was a major concern [5]. Indeed, such unprotected anal sexual acts are a recipe for heightened HIV-infections.

Forced FAS was also reported although to a smaller extent. As it is for the rape cases, forced FAS puts victims at a higher risk than those who bargain since the likelihood of using preventive measures is minimal or almost non-existent and is dependent on the HIV-awareness of the initiator. It is surprising to see that some of the males were forced by their guardians to practise FAS, implying more education is needed. Similar scenario of forced sex in the first anal sex practice featured in Ethiopia by 44% of the youths interviewed [5].

In this study, despite a good number of individuals having multiple partners, the use of condoms was found to be quite low for those engaged in FAS. The benefits of using condoms are well- documented especially in reducing the risk of HIV and other sexually transmitted infections, and cervical and penile associated lesion when used correctly and consistently [24, 25]. However, condoms usage is not widely used despite the Tanzania government and its development partners having spent a lot of resources on educational campaigns about condom use. Several reasons were provided for non-use of condoms, the main one being inconvenience in inserting penis into the anus. They said that the anus is dry by nature, hence making it doubly difficult to use condoms. Moreover, they perceived the anus to have more heat than the vagina, which made the condoms wear and tear during friction, particularly in the absence of lubricants. These findings are in line with a study carried out among university students in the United States [26].

Unprotected receptive anal intercourse is believed to be at least 10 times more risky than unprotected receptive vaginal intercourse for HIV infection and transmission [6, 27] and the risk is 20 times higher if the rectal lining is having other infections. The study findings reveal differences on the level of awareness of risks of acquiring HIV infection through FAS. Respondents in Makete and Siha districts perceived to have less risk of acquiring HIV infection through FAS than their counterparts in Kinondoni and Tanga districts. For those who believed they had less risk of HIV infection through unprotected anal sexual heterosexual intercourse several perceptions and misconceptions were linked to those beliefs. The common sentiments were that the anus is so dry that the virus cannot stay alive there for long as it is for the vagina; that the anus is used by fewer people hence less chances of coming into contact with HIV/AIDS-infected individuals. And yet, these very misconceptions make them even more vulnerable as they give them false assurance to practice unprotected anal sexual intercourse. Similar findings have been reported from other studies [5, 9, 28]. The study by Gross further revealed that anal sex was associated with non-use of condoms, having used narcotic drugs in the previous year and having a primary male partner [27].

Despite less preference for the use of condoms, only a small proportion has performed HIV testing. A good proportion of individuals tested for HIV were compelled to do so following the death of their partners, which indicates that more health education targeting behavioural change is still important.

On the other hand, the use of lubricants has been documented to reduce pain and make anal sex more comfortable [16] In another study, approximately one-fifth of women engaged in penile-anal sex during the previous month, 84.8% of them had used lubricants. Most the anal sexual intercourse with lubricant were rated as very pleasurable for more than 80% [29]. In this study several lubricants as well as substances such as soaps were mentioned to have been used to facilitate FAS. A popular lubricant mentioned include K-Y jelly which has been cited as expensive hence only affordable to a minority of the respondents who are practising FAS. The implication is that the majority resorted to using cheaper oils and other substances such as soaps and saliva. It is important to note that because of the delicate nature of the soft tissues in the anus, use of soaps poses a significant risk of HIV transmission. Other lubricants have not been tested for toxicity and safety, hence posing more health risks to the individuals than just the issue of HIV infection. Despite the widespread availability and use of lubricants, some studies have revealed that women and their partners question the safety of different types or brands of lubricants, the extent to which they may make sex feel more comfortable or pleasurable or whether certain lubricants may pose an increased risk of yeast or bacterial infections [30, 31]. Other studies have reported that the use of chemicals for lubricating the anus has been found to damage the cells and make them more susceptible to infection since rigorous laboratory tests have established that many of the lubricant products were toxic to cells and rectal tissues [16]. Considering the untested nature of substitutes found to be used in the current research, our study findings call for studies on chemical composition of the mentioned lubricants and substances, and their attendant health risks.

Studies of heterosexual HIV transmission have consistently found anal intercourse to be a highly predictive risk factor for sero-conversion [32]. For example, from 2001 to 2003, of the 157,252 persons diagnosed with HIV/AIDS in America, nearly 71% were men of which male-to-male sexual contact was the primary route of infection by 61% [34]. Anal sex was also found to be a common practice among sex workers along the truck stops and this was ironed out as an independent risk factor for HIV infection. Anal sex has been found to be associated with a higher risk of HIV infection in women [7]. Yet most HIV prevention messages targeting heterosexuals often focus on vaginal sexual intercourse and ignore anal sexual acts that could occur among these same heterosexual couples, presumably because of cultural taboos against acknowledging this sexual practice. In consequence, these massages continue to emphasise vaginal and, increasingly oral sex transmission. Inevitably, the health risks of anal sex among heterosexual partners have been severely underestimated by a substantial proportion of sexually active women and men in different parts of the world [32]. The risks of anal sex were also documented in a study by Maswanya et al. [14]. These sexual acts were mentioned by some of the men engaging in MSM.

## Study limitation

This study had to contend with one major limitation. Anal sex is not culturally and morally accepted in Tanzania so there might be cases of under-reporting of the prevalence of FAS among the respondents as the topic is culturally a sensitive one and even taboo among some social circles that could attract social stigma. In this regard, even the accuracy of self-reported cases may be questionable as there was no physical examination of the anal part. However, attempts were made to minimise this bias by ensuring confidentiality, privacy and anonymity throughout the study period and the reporting process. Despite this limitation, the study has a relatively larger sample and has used a combination of methods in a triangulated fashion in a bid to allow for the generalisation to other parts of Tanzania sharing similar characteristics with the districts under study. The implication is that the study results cannot be generalised to other parts of this East African region without proper qualification.

## Conclusions

Although the reported magnitude of FAS in this study as well as other previous studies conducted in Tanzania is relatively low, the actions surrounding the practice are associated with increased risks of HIV transmission. Generally, variations of FAS practices exist among the districts under review, with Makete having a higher proportion of individuals who engaged in FAS than other districts. The anal sex practice in heterosexual relationships is also associated with low use of condom and multiple sexual partners, hence putting individuals at high risk of HIV infection and transmission. There is, therefore, a need to carry out educational campaigns to educate all age groups on the inherent risks factors of FAS in this era of HIV/AIDS and its associated practices towards HIV infection. Furthermore, exploratory studies should be carried out to determine and document drivers of FAS.

## Additional file

**Additional file 1.** A questionnaire used in the data collection.

## Abbreviations

HIV: human immunodeficiency virus
AIDS: acquired immune deficiency syndrome
FAS: female anal sex among heterosexual couples
STIs: sexually transmitted infections
MSM: men having sex with men
CSWs: commercial sex workers
FGDs: focus group discussions
