# Supplementary material for: Prevalence and risk factors associated with female anal sex in the context of HIV/AIDS in the selected districts of Tanzania
Source: BMC Res Notes. 2017 Mar 27;10:140. doi: 10.1186/s13104-017-2452-9 (PMC5368909; doi:10.1186/s13104-017-2452-9)
Supplement: Supplementary file 1 — Additional file 1. A questionnaire used in the data collection. [file 13104_2017_2452_MOESM1_ESM.pdf]

QUESTIONAIRE

Prevalence, factors and health risks associated with female anal sex (FAS) in the context of HIV/AIDS Transmission in Tanzania

Family Information

1. Region____ ID.No.

2. District____ ID.No.

3. Ward____ ID.No.

4. Village/street____ ID.No.

5. Hamlet____ ID.No.

6. Head of the family ________________________________ ID.No.

7. Location(*1=Rural, 2=Urban)*……………………………………………………………………………………

8. Number of permenent household members…………………………………………………………………….

9. Number of household members aged 15 year and above Females ……………… Males

A: SOCIAL INFORMATION

(1) Age of the interviewee (*complete years*)……………………………………… ……….

Date of birth……………………………………………………………………… _____/_____/________

(2) Sex of the interviewee…………………………………………… *1=Female,2= Male*

(3) Marital status ………………………………………………………………………………

1= Not married
2= Married
3= Living together
4= Widow/widower
5= Separated
6= Divorced

(4)If the answer is 2 in the question above, what type of marriage? ……………………
1= One wife
2= More than one wife

(5) How old where you at the time of marriage? ....

(6) Do you have children?
……………………………………………………………………………………………..….

1=Yes
2= No

(7) *If yes, mention number of children*…………………………………………………………

(8) What is your religion ?
……………………………………………………………………………………..

1= Christian
2=Muslim
3= No religion
*4= Other ,mention*________________________________

(9)What is your highest level of education? ....

1= No education
2= Adult education
3= Primary education
4= Secondary education
5=College (certificate/diploma)
6= University education(1st or 2nd degree)

(10) What kind of income generation activity you are involved in ?..............................

1= no specific activity
2= Farming
3= Government employee
4= Business
5= Student
6= Other ,mention ___________________________

(11) How long have you stayed in this community ……………………………..……….

B: History on Sexual behavior

(12) Have you had a sexual partner(s)?…………………………………………………….

1= Yes
2=No

(13) *If yes, How many ?*
……………………………………………………………………………………………..

99……… No answer/ don't remember

(14) Currently, do you have a permanent partner?…………………………………………………………

1=Yes
2=No

(15) Have you had a temporally partner? …………………………………………………………….

1= Yes
2= No →***Go to Qns25***

(16) If yes, how many?

……………………………………………………………………………………………………..

99……… No answer/ don't remember

(17) Currently, do you have a temporary partner(s)?……………………………………………………………………

1. *Yes*
2. *No→**Go to Qns25***

(18) Have you had sexual intercourse with temporary partner(s)?/How many?/did you use any condom? Je, ***(Probe any type of sex which was conducted, multiple answer are allowed .)***

	21. How many temporary partner(s)	22. Did you use condom ? ***(1=Yes, 2=No)***
1. Normal sex (inserting penis in the vagina)		
2. Having sex with men (Inserting penis in anus of a man)		
3. Sex with female to female (lesbianism)		
4. Having anal sex with a woman (Inserting penning in anus) of a woman		
5. Sex at the mouth (inserting penis in the mouth of a partner)		
6. Others ,mention ________________________________		

19) What reasons motivated you to use condom when you had sex ? ***(Probe, multiple answers are allowed)***

1. *To prevent HIV transmission*
2. *To prevent pregnancy*
3. *Family planning*
4. *To increase pleasure*
5. *Showing you partner that you care*
6. *I don't know*
7. *Othesr,mention*____

(20) Have you ever heard of sex female anal sex?(Inserting penis in anus of a woman)

1. *Yes*
2. *No*

(21)Have you been convinced to have anal sex (Inserting penis in (anus) ***(Note the sex of the interviewee when asking this question)***

1. *Yes*
2. *No*

(22) If yes, who convinced you to have anal sex at ***(Note the sex of the interviewee when asking this question)…. (Probe, multiple answers are allowed)***

1. Permanent partner (friend)
2. Permanent partner(husband/wife)
3. Temporary partner
4. Family member
5. House maid
6. My boss
7. Other,mention____

(23) When you where convinced to have anal sex, did you agree?……………………………

1. *Yes*
2. *No*→***Go to Qns36***

(24) What motivated you to have anal sex after you have been convinced/intimidated? ------- ***(N[illegible]e one who was mentioned in the above question)***

1. To get money
2. To get pleasure
3. To maintain the culture
4. Avoid pregnancy to a partner
5. Test what you hear
6. Witchcraft beliefs
7. I had no option
8. Other reason
   mention____

(25) **FOR FEMALE ONLY :** Have you been forced (raped) to have anal sex by a man? (Inserting penis in anus) of a woman)

1. *Yes*
2. *No*

(26) FOR FEMALE ONLY**:** Who forced you (rape) to perform anal sex

1. Permanent partner (friend)
2. Permanent partner(husband/ wife)
3. Temporary partner
4. Family member
5. House help
6. My job supervisor
   Others, specify____

(27) What actions are usually done before engaging in anal sex? (Probe; answers may be more than one, circle what ever mentioned)

1. Sexual arousal actions (caress, normal sex, watching pornography, …)
2. Cleaning rectum
3. Use of alcohol such alcoholic drinks, illicit drugs, etc.
4. Others, specify ____________________________

(28) Do you know any item that is used when performing anal sex?

1. *Yes*
2. *No*

(29) If Yes, what are those items and why they are used?

**Item Used**	**Reasons**
1.	1.
2.	2.
3.	3.
4.	4.
5.	5.

(30) Do you think there are advantages of using condoms when performing female anal sex.... (Probe: the answer may be more than one, circle what ever mentioned)

1. Preventing the transmission of STIs or HIV / AIDS
2. Make it easier to insert the penis in the rectum
3. Reduce pain when inserting the penis
4. Prevent penis from fecal smear
5. Do not know / do not remember
6. Other, specify____________________________

THE FOLLOWING QUESTIONS refers to those who engaged in female anal sex

(31) For the past one year how often have you had female anal sex?

(32) In the past one year how many sexual partners did you have in female anal sex practices

(33) When you engaged in anal sex did you use a condom?

1. *Yes*
2. *No*

(34) Did you use condoms the last time you engaged female anal sex ? (Inserting the penis into the anus

(35) If yes, what reasons made you to use a condom?.... (Probe: the answer may be more than one, circle what ever mentioned)

1. Prevention of sexually transmitted diseases or HIV / AIDS
2. Make it easier to insert the penis in the rectum
3. Prevent penis from fecal smear
4. I do not know
5. Other, specify_______________

(36) If you did not use a condom what reasons made you not to use?.... (Probe: the answer may be more than one, circle whatever mentioned)

1. Condoms reduce pleasures
2. Penis fail to erect
3. It shows you don't trust your partner
4. It may rupture or tear
5. Not Found
6. I do not know
7. Other, specify_____________________

(37) Do you feel you are at risk of getting HIV infection when having anal sex? ☐

1. *Yes*
2. *No*

(38) If yes, do you think you're at risk of which level of infection HIV when having anal sex (Read the answers) ☐

1. *Highest level*
2. *Normal level*
3. *Lower level*
4. *No chance of catching HIV*
5. *I have HIV infection ALREADY*
6. *I'm not sure*

(39) Let me know if you have ever tested for HIV? (*Tell her no need to know the answers to HIV tests*) ☐

1. *Yes*
2. *No*

(40) If yes, what reasons made you test for HIV ……………..………………………………………. ☐

1. *Advised by a physician or health care provider*
2. *Recurrent illness*
3. *Know my health status*
4. *During pregnancy*
5. *Encouraged - during the campaign*
6. *Marriage*
7. *Partner died after a long illness*
8. *Other a (specify*

(41) If No, What reasons made you not to test for HIV……………………………………………………………..………

1. Religious Beliefs
2. No HIV test services in the nearby facility
3. Do not trust Health care providers (confidentiality, privacy)
5. Fear
6. Stigma
7. Feel Safe
8. Do not trust the HIV test

(42) Do you think what can be done to prevent / reduce transmission of HIV to people who engaged in female anal sex in Tanzania?

____

End of interview

1. Thank the respondent for her/his time
